# Optimization, evaluation, and comparison of standard algorithms for image reconstruction with the VIP-PET

**DOI:** 10.1088/1748-0221/9/07/C07004

**Published:** 2014-07

**Authors:** E. Mikhaylova, M. Kolstein, G. De Lorenzo, M. Chmeissani

**Affiliations:** Institut de Física d’Altes Energies (IFAE), Universitat Autónoma de Barcelona (UAB), 08193 Bellaterra (Barcelona), Spain

**Keywords:** Medical-image reconstruction methods and algorithms, computer-aided software, Gamma camera, SPECT, PET PET/CT, coronary CT angiography (CTA)

## Abstract

A novel positron emission tomography (PET) scanner design based on a room-temperature pixelated CdTe solid-state detector is being developed within the framework of the Voxel Imaging PET (VIP) Pathfinder project [1]. The simulation results show a great potential of the VIP to produce high-resolution images even in extremely challenging conditions such as the screening of a human head [2]. With unprecedented high channel density (450 channels/cm^3^) image reconstruction is a challenge. Therefore optimization is needed to find the best algorithm in order to exploit correctly the promising detector potential. The following reconstruction algorithms are evaluated: 2-D Filtered Backprojection (FBP), Ordered Subset Expectation Maximization (OSEM), List-Mode OSEM (LM-OSEM), and the Origin Ensemble (OE) algorithm. The evaluation is based on the comparison of a true image phantom with a set of reconstructed images obtained by each algorithm. This is achieved by calculation of image quality merit parameters such as the bias, the variance and the mean square error (MSE). A systematic optimization of each algorithm is performed by varying the reconstruction parameters, such as the cutoff frequency of the noise filters and the number of iterations. The region of interest (ROI) analysis of the reconstructed phantom is also performed for each algorithm and the results are compared. Additionally, the performance of the image reconstruction methods is compared by calculating the modulation transfer function (MTF). The reconstruction time is also taken into account to choose the optimal algorithm. The analysis is based on GAMOS [3] simulation including the expected CdTe and electronic specifics.

## 1 Introduction

Details about the design, geometry, electronics and the simulation of expected performance of the VIP scanner based on pixelated CdTe detectors is presented in [2]. Simulation results indicate that the VIP design has the potential to provide a clinical head scanner with combination of excellent spatial (~ 1 mm FWHM) and energy resolutions (1.6%) [4]. The crack-free geometry of the VIP scanner with 4-cm-thick detectors provides a sensitivity of 14.37 cps/kBq (according to the NEMA NU 2-2001 standard [5]). A very low scatter fraction of 3.95% (according to the NEMA NU 2-2001 standard [5]) is achieved due to the good energy resolution provided by the CdTe detectors. The high number of channels in the VIP scanner makes the full system less affected by the dead time of the individual detector voxels. The [2] showns that the VIP allows to dramatically shorten the scan time and thereby lower the image blurring due to the motion of patients. Thus, the patient dose can be significantly reduced while keeping the time of the screening the same. Moreover, images acquired with the VIP are generally characterized by high contrast and low noise, and are obtained with a relatively small number of coincidences.

However, even if the VIP scanner is capable to obtain a high-purity sample, the final image quality depends on the image reconstruction method and the reconstruction parameters being used. The use of an inappropriate reconstruction algorithm can distort the final image, lowering its quality and adding artifacts. On the other hand, a suitable method can produce a high quality image even if the collected data is noisy. The choice of reconstruction technique is one of the crucial factors in getting a good final image. For this reason the designers of PET systems often develop their own algorithm (or modify existing algorithms) to adapt it to the specifics of the scanner. In case of the VIP PET scanner, we test and evaluate a few standard methods to pick the optimal one in terms of image quality and reconstruction time.

## 2 Detector specifications and simulation setup

**Figure 1:**
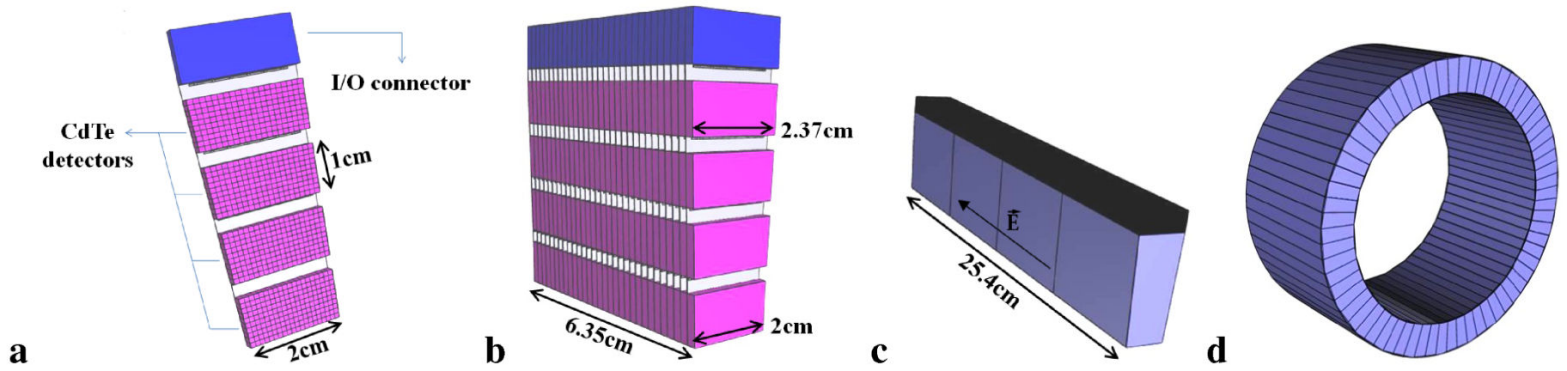
**a:** the VIP detector module; **b:** a VIP module block consists of 30 detector modules; **c:** a VIP ring section formed from 4 module blocks; **d:** the VIP scanner that is made of 66 VIP ring sections.

The VIP scanner has a modular design based on the detector module shown in (figure 1a). The VIP detector module has 4 CdTe detectors each with dimensions of 10 mm × 20 mm × 2 mm. Each of them is electronically pixelated into 200 voxels of 1 × 1 × 2 mm^3^ pitch, for an accurate photon impact point measurement, and bonded to a thinned read-out channel (ROC) and then mounted on a kapton printed circuit board (PCB). A distinctive characteristic of the VIP is that the module can be given a trapezoidal shape to form a scanner ring without cracks to boost the system sensitivity. In the proposed design, incident radiation traverses a minimum of 4 cm CdTe with 70% of singles 511 keV photons being completely absorbed. The coincidence time resolution of two equal 2 mm thick CdTe detectors, analogous to the ones to be employed in the VIP system, was measured in [6]. Results show that a 20 ns coincidence time window corresponds to a detection efficiency of 70% photon pairs. The VIP module block consists of 30 detector modules stacked together (figure 1b). Thereafter, 4 such module blocks connected to the same electronic bus form a VIP section (figure 1c). Finally, when 66 sections are put together, they form a cylindrical seamless PET scanner (figure 1d) with a total of 6,336,000 detector voxels. The complete scanner has an inner diameter of 42 cm, an outer diameter of 54 cm, and an axial length of 25.4 cm to match the typical size of a brain PET. Due to the large number of individual channels, the design of the electronics for the signal processing and readout is a crucial for VIP. The signal processing takes place “in-situ” with the application-specific integrated circuit (ASIC) bump-bonded to the detector voxels. The ASIC design, the development status, and the results of the successful characterization of the preliminary prototypes are described in detail in [7].

In order to evaluate the VIP system performance and assess its image quality, the whole VIP geometry is simulated using the Geant4-based Architecture for Medicine-Oriented Simulations (GAMOS) [3]. The Geant4 kernel provides the accurate simulation of particle interaction with the materials within the defined geometry.

## 3 Analysis

Four different image reconstruction methods are considered. The first is the 2-D Filtered Back-projection (FBP) reconstruction algorithm with the single-slice rebinning technique (SSRB) [8], probably the most commonly used algorithm in tomographic images. The 2-D FBP is an analytic method and therefore it is very fast. The second one is the Origin Ensemble (OE) algorithm [9]. The OE is a stochastic and relatively fast converging technique. The speed of convergence does not depend on the number of channels. The next method is Ordered Subset Expectation Maximization (OSEM) [10]. It is an iterative algorithm and thus computationally more intensive than FBP and OE and therefore considerably slower. One of the advantages of iterative reconstruction techniques is that they are less sensitive to the imperfections of the detector and the dataset. Finally, the last method considered in this analysis is the List-Mode OSEM (LM-OSEM) [11, 12]. It is also an iterative algorithm but, unlike OSEM that is based on the definition of a system matrix mapping the probabilities of all the possible combination of data for a given field of view (FOV), LM-OSEM only considers the detected events that are presented in list mode. The “a-posteriori” approach reduces considerably the amount of memory needed by OSEM to map the system matrix. In order to evaluate the performance of each reconstruction method we used three different modalities: the bias, the variance and the mean square error (MSE) measurements [13]; calculation of the modulation transfer function (MTF) curve [14]; and the region of interest (ROI) analysis [15].

### 3.1 Optimization of the reconstruction parameters

One can find the best reconstruction parameters and compare performance of various reconstruction techniques using image quality metrics such as the bias, the variance, and the average MSE. The bias indicates how much difference is generated between the group average image and the true image [13]. The variance is a measure of how consistent the several reconstructed images are. It compares each image of the sequence with the group average image [13]. These two merits provide a useful comparison of the reconstruction methods, however they do not indicate the optimal configuration of the reconstruction parameters to obtain the best quality image. The MSE value combines the bias and the variance values (*MSE* = *variance* + *bias* * *bias*) in such a way, that retrieving the minimum MSE value is equivalent to having the optimal pair between variance and bias parameters. The MSE measures the difference between a reconstructed image and the true image [13]. The average MSE is the arithmetic mean of all the MSE for a given image set. Varying the parameters of the different algorithms and comparing these image quality metrics one finds the optimum reconstruction parameters for each of the algorithms. We consider the best image is the one that gives the lowest value of the average MSE.

**Figure 2:**
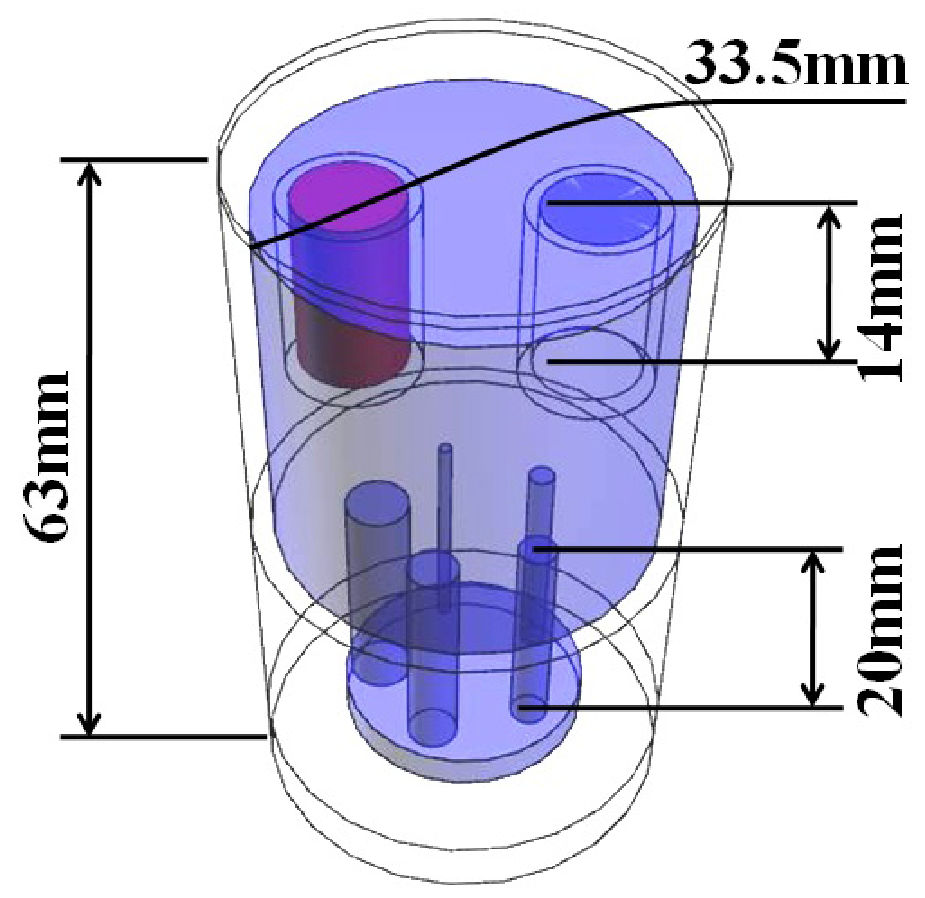
NEMA NU 4-2008 phantom.

For the quality metrics calculation we chose a small-animal PET phantom described in NEMA NU 4-2008 protocol (figure 2) [15]. The phantom is made of polymethylmethacrylate and has internal dimensions of 50 mm in length and 30 mm in diameter. It consists of three parts: two lids and the main body. The main body has 5 drilled through rods of 1, 2, 3, 4, and 5 mm in diameter, respectively. Their length is 20 mm. The top lid has two cylindric chambers of 15 mm in length and 8 mm in diameter. The main body’s chamber, as well as the rods, are filled with ^18^F radioactive water of 3.7 MBq total activity. One of the top lid’s chambers is filled with air and the other one is filled with non-radioactive water in order to create two cold regions.

### 3.2 MTF test

The performance of the reconstruction algorithms can be evaluated by comparison of the MTF curves obtained for each method. Computation of the MTF is one of the most complete methods to characterize the spatial resolution of a scanner in tomography. Good low-frequency response better represents large low-contrast lesions, while good high-frequency response is better for fine details and sharp edges [16]. The MTF was calculated by taking the 1-D Fourier transform (FT) of a line profile recorded through the center of the point spread function (PSF) in the direction of the profile. It is a good approach (respect to 2-D MTF from 2-D FT of the 2-D PSF or a 3-D MTF from a complete 3-D data set for the PSF) when a reconstructed point-like source has a symmetrical shape in all directions. In case of the VIP scanner such good symmetry is reached if the point-like radiation source is placed near the center of the axial and transaxial FOV of the system [2].

To obtain the PSF we simulated an ideal 511 keV back-to-back gamma point-like source of low activity. It was placed in the center of the axial FOV and at 5 mm off the center of the transaxial FOV in order to avoid a central artifact. The images of the point-like source were reconstructed using the optimal number of iterations (found in the section 4.1) for each iterative reconstruction method. In case of the 2-D FBP algorithm no smoothing was used in order to not loose the contrast. After the reconstruction the MTFs were computed. As a general rule the smaller image pixel size the better spatial resolution can be reached by a scanner and, thus, the more detailed image can be obtained. However, increasing the voxel density in the FOV corresponds to reducing the statistics per voxel with the effect of increasing the image noise. A pixel size of 0.25 mm is chosen to fulfill the sampling requirement for an imaging detector presented in [16]:

(3.1) $$\Delta r\leq \frac{FWHM}{3}$$

where Δ*r* is the chosen pixel size, FWHM is the full-width at half-maximum of the PSF of the scanner (~0.8 mm in the center of the FOV according to [2]).

### 3.3 The image quality evaluation. ROI analysis

Once the best reconstruction parameters are chosen (for each reconstruction technique) based on the results shown in the subsection 4.1, the ROI analysis is performed. According to the NEMA NU4-2008 prescriptions a 22.5-mm-diameter (75% of the total diameter) by 10-mm-long cylindric volume of interest (VOI) was drawn over the center of the uniform region of the image quality phantom. The average activity concentration, the maximum, and the minimum values in the VOI, and the percentage standard deviation (%SD) were calculated to assess the signal to noise ratio performance. In order to compute the contrast, the reconstructed image slices covering the central 10-mm length of the rods were averaged to obtain a single slice of lower statistical noise. The image contrast is related to values of recovery coefficients (RC) in the hot regions and to the spill-over ratio (SOR) in the cold areas. The RC is the measured activity concentration divided by the actual activity concentration. The NEMA NU-4 2008 standard calculates RC values for each hot rod by dividing the mean activity concentration along the 10-mm line profile of each rod by the average activity concentration of the uniform region. The SOR is the mean activity concentration in the cold regions (water- and air-filled cylindric inserts) relative to the mean activity concentration in the hot uniform region.

## 4 Results

### 4.1 Optimization of the reconstruction parameters

A total of 10 million coincidence sinograms were collected to reconstruct the image. No attenuation, scatter or random corrections nor normalization were applied for any reconstruction algorithm. The image pixel size is 0.25 mm and the slice thickness is 2 mm. The bias, variance and average MSE were measured for sets of 10 images. The images were reconstructed from 10 data sets (10 collections of LORs) with a single varying reconstruction parameter for each method.

**Figure 3:**
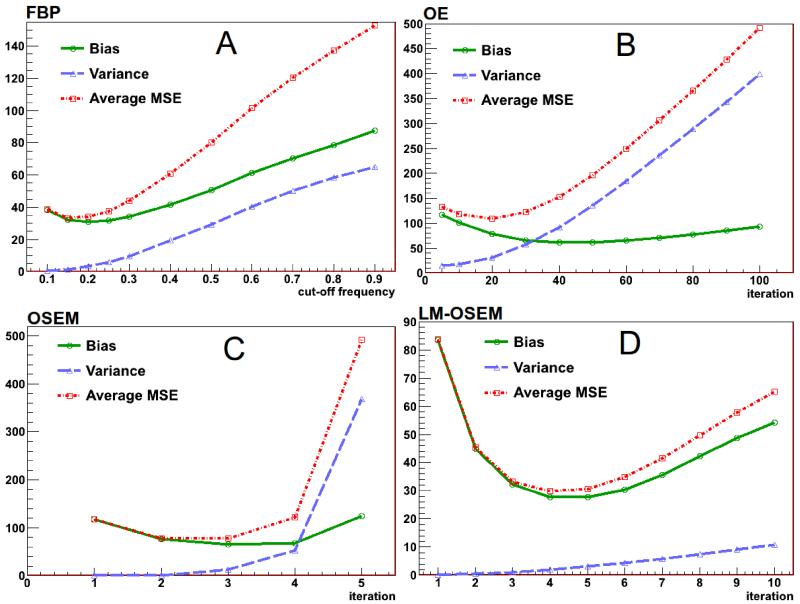
Image quality metrics versus: **A:** cutoff frequency for FBP with a Hamming filter, **B:** number of iterations for OE, **C:** number of iterations for OSEM, **D:** number of iterations for LM-OSEM.

**Figure 4:**
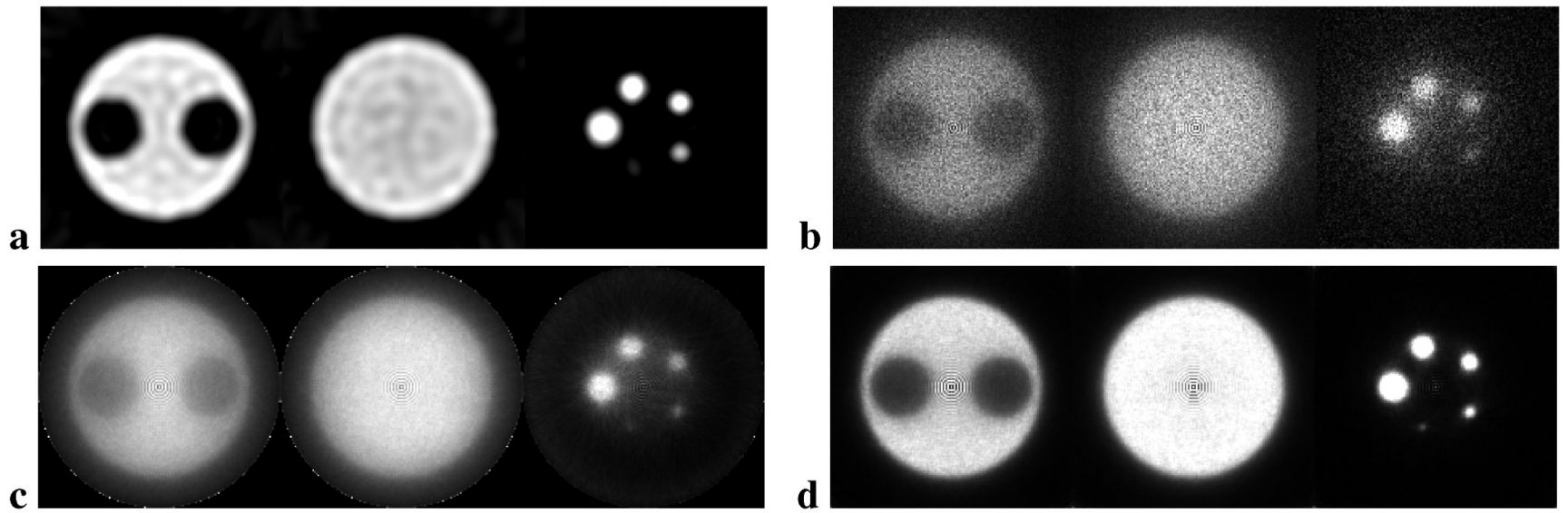
**a:** FBP reconstructed image with a Hamming filter (cutoff frequency = 0.15). **b:** OE reconstructed image after 20 iterations (no filters). **c:** OSEM reconstructed image after 2 iterations and 2 subsets (no filters). **d:** LM-OSEM reconstructed image after 4 iteration and 2 subsets (no filters).

The cutoff frequency relative to Nyquist frequency for the Hamming filter [17] was varied for every run of the 2-D FBP algorithm. The result is shown in figure 3a. As one can see the optimal value for the cutoff frequency is 0.15, where the average MSE has the lowest value (33.5352). The OE reconstruction method was tested by varying the number of iterations. The result is shown in figure 3b. The minimum value for the average MSE (109.21) corresponds to the image reconstructed after 20 iterations. The number of iterations was also varied for the OSEM reconstruction. Each time 2 subsets were used. The resulting bias, variance and average MSE are shown in figure 3c. The average MSE shows a minimum value (76.84) for 2 iterations. Finally, the LM-OSEM algorithm was tested varying number of iterations and using 2 subsets for each run. The result is shown in figure 3d. The minimum average MSE has a value 29.7853 for 4 iterations. The images obtained with optimal parameters for each of the algorithms are shown in figure 4.

### 4.2 MTF test

**Figure 5:**
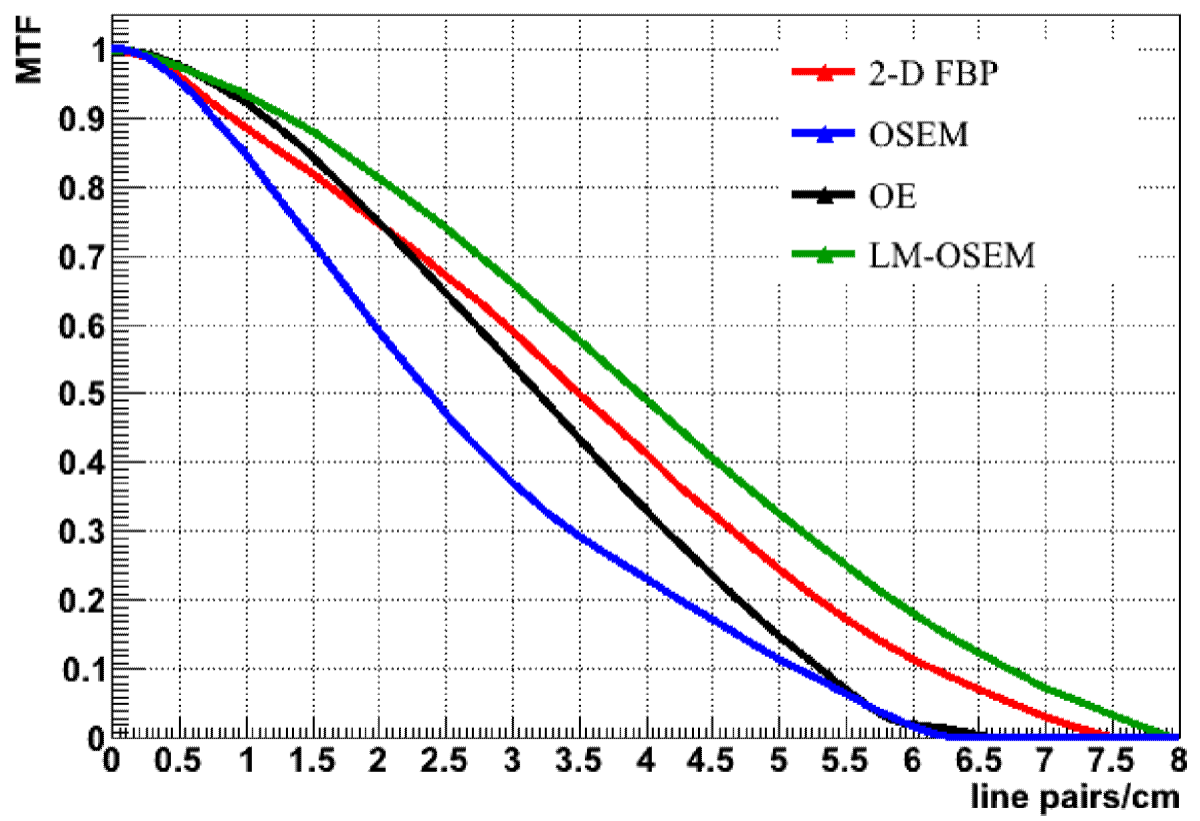
The MTFs obtained from the reconstructed PSF.

The results on the measurement of MTFs are presented in figure 5. As one can see the LM-OSEM method with 4 iterations and 2 subsets shows the best MTF. 2-D FBP (no smoothing) and OE (after 20 iterations) have similar performance. OE is a bit better for the reconstruction of coarse and low-contrast objects, and 2-D FBP is more suitable for the representation of fine detail. OSEM shows the worst results (after 2 iterations and 2 subsets) because of the lack of computer memory to handle the probability distribution for 6,336,000 detector voxels, so that the voxels had to be merged to reduce the multiplicity.

### 4.3 The image quality evaluation. ROI analysis

The ROI analysis was performed on the images with the best quality of each reconstruction technique (figure 4). The results are summarized in table 1. The images reconstructed with the 2-D FBP and the LM-OSEM methods have the highest contrast. Additionally, the 2-D FBP algorithm gives the lowest level of noise.

## 5 Conclusion

A comparison of performance of 4 standard image reconstruction methods for the VIP scanner was performed. The optimal reconstruction parameters for each algorithm and for the given image voxel size (0.25 mm × 0.25 mm × 2 mm) were found. An evaluation of quality of the images reconstructed with these parameters was performed in accordance with the NEMA NU-4 2008 standard. The NEMA NU-4 2008 small animal phantom was chosen for the tests. The results of all tests are summarized in table 2. The whole analysis shows that the best techniques to reconstruct the VIP images are FBP and LM-OSEM. Both give comparable high contrast. However, in terms of noise level and reconstruction time consuming, FBP shows better performance than LM-OSEM. The LM-OSEM reconstruction provides slightly better spatial resolution and thus provides images with more detail. Additionally, to get the high quality images it does not require a big number of iterations. The OE and the OSEM algorithms produce images of inferior quality when compared to FBP and LM-OSEM. In this case additional optimization and the data correction are necessary to obtain better quality images. The MTF test results (for the image pixel size of 0.25 mm) are in good agreement with the spatial resolution values published in [2].

## Figures and Tables

**Table 1:** Quality parameters comparison for the phantom reconstructed by different algorithms.

Parameter	FBP cut-off = 0.15	OE 20 iterations	OSEM 2 iterations	LM-OSEM 4 iterations
RC(%STD) 1 mm	0.3(12.7%)	0.08(86.1%)	0.053(19.7%)	0.28(22.1%)
RC(%STD) 2 mm	0.796(11.1%)	0.12(98.6%)	0.088(22.8%)	0.8(23.4%)
RC(%STD) 3 mm	1.2(10.1%)	0.16(89.2%)	0.14(19.4%)	1.0(26.8%)
RC(%STD) 4 mm	1.2(10.2%)	0.26(77.9%)	0.2(20.5%)	1.0(26.7%)
RC(%STD) 5 mm	1.19(10.8%)	0.25(76.8%)	0.17(20.8%)	0.85(29.8%)
Uniformity max.	4.66	169	0.0117	284
Uniformity min.	2.08	3	0.002	6.34
Uniformity mean	3.44	54.28	0.004	34.6
Uniformity %STD	9.74%	43.7%	10.5%	16.99%
SOR(%STD) water	0.025(18.9%)	0.4(96.9%)	0.51(12.6%)	0.22(21.4%)
SOR(%STD) air	0.035(19.7%)	0.38(54.8%)	0.5(14.9%)	0.22(18.2%)

**Table 2:** Reconstruction algorithms comparison.

	2-D FBP cut-off = 0.15	OE 20 iterations	OSEM 2 iterations	LM-OSEM 4 iterations
Minimum average MSE	33.5	109.2	76.84	29.8

at MTF = 0.5	3.5 lp/cm	3.2 lp/cm	2.38 lp/cm	3.95 lp/cm
corresponding line width	1.43 mm (cut-off=1)	1.56 mm	2.1 mm	1.27 mm

at MTF = 0.1	6.15 lp/cm	5.32 lp/cm	5.15 lp/cm	6.725 lp/cm
corresponding line width	0.81 mm (cut-off=1)	0.94 mm	0.97 mm	0.74 mm

5 mm rod: RC(%STD)	1.19(10.8%)	0.25(76.8%)	0.17(20.8%)	0.85(29.8%)

CPU time	~1 min.	~36 min.	~6 min.	~47 min.
